# Testicular Pouch Hibernoma: A Case Report

**DOI:** 10.7759/cureus.20702

**Published:** 2021-12-26

**Authors:** Eric Martinez Lino, Waldomiro Camargo, Beatriz Varanda Rizzo Teixeira, Eduardo Sonego de Toledo, Ana Clara Nagle Spessoto, Maria Fernanda Warick Facio, Fernando Nestor Facio, Luís Cesar Fava Spessoto

**Affiliations:** 1 Urology, Faculty of Medicine of São José do Rio Preto, São José do Rio Preto, BRA; 2 Internal Medicine, Faculty of Medicine of São José do Rio Preto, São José do Rio Preto, BRA; 3 Medicine, Medical School of Catanduva, Catanduva, BRA; 4 Integrative/Complementary Medicine, Faceres Medical School, São José do Rio Preto, BRA

**Keywords:** neoplasms, lipoma, scrotum, testicular pouch, hibernoma

## Abstract

Hibernomas are rare lipomatous tumors derived from brown adipose tissue. Only two cases of hibernomas in the scrotum have been reported in the literature so far. Brown adipose tissue is responsible for thermogenesis in hibernating mammals and embryos. In adult humans, reminiscent brown tissue is most frequently located in the axial skeleton, scapular waist, and neck. This case report describes the finding of a testicular pouch hibernoma in a 34-year-old male who presented with a nodule in the scrotum, which was initially suspected to be a lipoma. The diagnosis was confirmed by histopathological analysis, and the patient was treated by surgical excision. Despite its rarity, hibernoma should be part of the differential diagnosis for lipoma, the most frequent benign mesenchymal neoplasm worldwide.

## Introduction

Hibernomas are benign tumors derived from residual brown adipose tissue. Unlike lipomas, they are extremely rare, with less than 200 reported cases in the literature. Hibernomas most often occur proximally to the axial skeleton, where brown fat existed in the fetus and has persisted into adulthood [1]. So far, only two case reports of tumors derived from brown adipose tissue in the scrotum have been published in the literature. In this report, we present a rare case of a patient with testicular pouch hibernoma.

## Case presentation

A 34-year-old male from São José do Rio Preto (São Paulo, Brazil) presented with a gradual increase of scrotal volume in the course of the last six years. There were no other associated signs or symptoms. He was taking escitalopram for anxiety. He had no history of previous surgeries. On physical examination, the right testicle showed a 2-cm nodule; it was superficial, mobile, fibroelastic, and painless.

**Figure 1:**
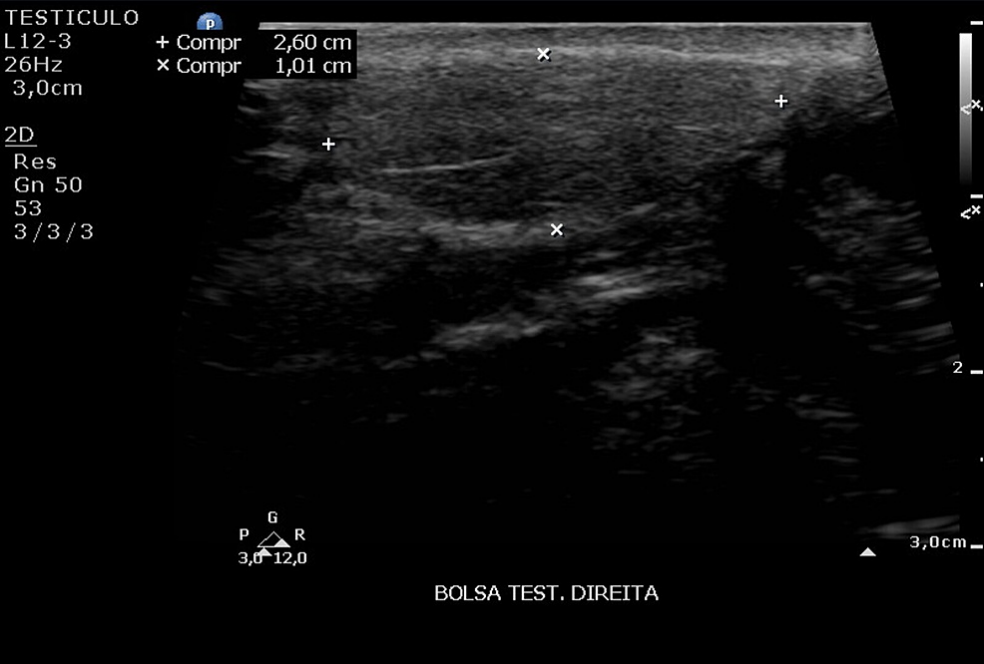
Ultrasound of the right testicular pouch Presence of nodular image, measuring 2.6 x 2.1 x 1.0 cm, isoechogenic to adipose tissue

A Doppler ultrasound was requested for further evaluation of the nodule. The testicles and epididymis had normal echographic aspect; a subcutaneous nodular image with regular contours and imprecise limits, isoechogenic to adipose tissue, was seen in the right testicular pouch (2.6 x 2.1 x 1.0 cm) (Figure 1).

**Figure 2:**
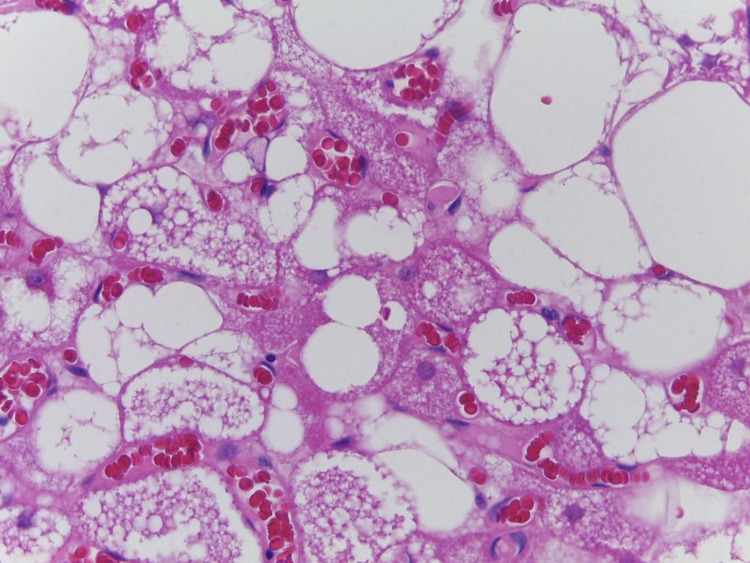
Histopathological examination Multivacuolated cells with granular eosinophilic cytoplasm, confirming the diagnosis of hibernoma

The diagnostic hypothesis was that it was a lipoma. The lesion was excised in the Urology outpatient clinic of Hospital de Base (São José do Rio Preto, São Paulo, Brazil) and sent for anatomopathological study with hematoxylin and eosin staining (Figure 2). The analysis revealed multivacuolated cells with granular eosinophilic cytoplasm. A diagnosis of scrotum hibernoma was made. The immunohistochemistry was not performed due to the coronavirus disease 2019 (COVID-19) pandemic. In this case, the excisional biopsy was both diagnostic and therapeutic, as the excision margins were clear. The procedure was performed with no complications and the patient was discharged on the same day. All procedures performed were in accordance with the ethical standards of the institutional and national research committees and with the Declaration of Helsinki (as revised in 2013).

## Discussion

Non-germinative testicular tumors account for approximately 5% of all testicular tumors and thus are rare among urological neoplasms [2]. In the testicular annexes, benign mesenchymal tumors (leiomyoma, fibroma, lipoma, and lymphangioma) are the most common types. Lipomas are the most frequent benign mesenchymal neoplasm worldwide. It usually presents in subdermal and subcutaneous tissues, but it can occur in any region of the body, including viscera and cavities [2,3].

In hibernating animals, brown adipose tissue is involved in the production of heat to maintain body temperature. It is highly vascularized, organized in clusters, and surrounded by white adipose tissue. It can be found in varying amounts among species and their lineages. In adult humans, reminiscent brown adipose tissue is generally located in the interscapular, intercostal, periaortic, perirenal, axillary, cervical, and ventral regions [3]. The capacity and activity of brown adipose tissue in animals' thermogenesis are adjusted by environmental conditions: it is atrophied or activated depending on the energy demands [3].

In adult humans, hibernomas are a rare brown adipose tissue-derived tumor. On physical examination, it presents as a superficial, mobile, fibroelastic subcutaneous mass. In the Doppler ultrasound exam, hibernomas manifest as solid, well-circumscribed hyperechoic masses with an increased central vascular flow [4].

The definitive diagnosis is made through histopathological examination with hematoxylin and eosin staining. It demonstrates three cell types: cells with granular eosinophilic cytoplasm containing lipid vacuoles; large cells containing scarce granular eosinophilic cytoplasm and multiple lipid vacuoles; and large uni-vacuolated adipocytes [2].

Fine needle aspiration biopsy is not recommended in suspected cases of hibernoma: due to its hypervascularity, there is a significant chance of bleeding. The treatment consists of complete excision of the lesion [5].

## Conclusions

Hibernoma is a rare benign tumor, with only two cases reported in the testicular pouch so far. Its growth is slow and painless, and previous cases have been mistaken for lipoma or liposarcoma. The definitive diagnosis is made with total excision of the lesion, followed by histopathological evaluation.
